# Development of a Foot-and-Mouth Disease Virus Serotype A Empty Capsid Subunit Vaccine Using Silkworm (*Bombyx mori*) Pupae

**DOI:** 10.1371/journal.pone.0043849

**Published:** 2012-08-27

**Authors:** Zhiyong Li, Yongzhu Yi, Xiangping Yin, Yun Zhang, Ming Liu, Hang Liu, Xuerui Li, Yinü Li, Zhifang Zhang, Jixing Liu

**Affiliations:** 1 State Key Laboratory of Veterinary Etiological Biology, Key Laboratory of Grazing Animal Diseases of Ministry of Agriculture, Lanzhou Veterinary Research Institute, Chinese Academy of Agriculture Sciences, Lanzhou, Gansu, China; 2 Biotechnology Research Institute, Chinese Academy of Agricultural Sciences, Beijing, China; Nanyang Technological University, Singapore

## Abstract

Foot-and-mouth disease (FMD) is a highly contagious disease of cloven-hoofed animals that inflicts severe economic losses in the livestock industry. In 2009, FMDV serotype A caused outbreaks of FMD in cattle in China. Although an inactivated virus vaccine has proven effective to control FMD, its use may lead to new disease outbreaks due to a possible incomplete inactivation of the virus during the manufacturing process. Here, we expressed the P1-2A and the 3C coding regions of a serotype A FMDV field isolate in silkworm pupae (*Bombyx mori*) and evaluated the immunogenicity of the expression products. Four of five cattle vaccinated with these proteins developed high titers of FMDV-specific antibody and were completely protected against virulent homologous virus challenge with 10,000 50% bovine infectious doses (BID_50_). Furthermore, the 50% bovine protective dose (PD_50_) test was performed to assess the bovine potency of the empty capsid subunit vaccine and was shown to achieve 4.33 PD_50_ per dose. These data provide evidence that silkworm pupae can be used to express immunogenic FMDV proteins. This strategy might be used to develop a new generation of empty capsid subunit vaccines against a variety of diseases.

## Introduction

Foot-and-mouth disease (FMD) is a highly contagious disease of livestock that causes severe economic loss in susceptible cloven-hoofed animals, such as cattle, swine, sheep, and goats. FMD virus (FMDV) is a positive-sense single-stranded RNA virus belonging to the *Aphthovirus* genus, family *Picornaviridae* [1]. The current FMD vaccine is a chemically inactivated whole virus preparation that has been used to effectively control the disease in enzootic countries but incomplete inactivation or escape of virus from manufacturing facilities during the preparation of the whole virus vaccine is a potential danger for new outbreaks [2]. It has been proven that the virus involved in FMD outbreaks was sometimes closely related to virus strains used in vaccine manufacturing plants [3]. In addition, since inactivated vaccines contain various amounts of contaminating viral non-structural proteins, it may be difficult to distinguish vaccinated from infected animals [4].

To overcome these issues, a number of different types of vaccines have been developed including synthesized peptide vaccines [5], [6], recombinant virus-vectored vaccines [4], [7]–[11], empty capsid subunit vaccines [12], [13], [14], DNA vaccines [15], [16], and an oral vaccine produced in transgenic plants [17]. Among these, the empty capsid subunit vaccine that contains all of the immunogenic sites present on the intact virions but is lacking nucleic acid is very safe and effective. Expression products from the baculovirus expression system are generally considered to be immunogenic and possess the ability to assemble into empty viral capsids [18]. In the baculovirus expression system, *in vitro* culture using cells is commonly used but insect larvae and pupae may also be used for protein production [19]. Moreover, protein expression levels in silkworm larvae and pupae have been shown to be 50–1000 times higher than for insect cell lines [20]. Compared to the silkworm larvae, the silkworm pupae are more convenient for large-scale production and can be used to express the protein at any time without the limitation of supplying mulberry leaves.

FMDV consists of seven serotypes, named O, A, C, Asia I, SATI, SAT II, and SAT III, where viral infection or vaccination with one serotype does not confer protection against the other serotypes [21]. In a previous report, we assembled empty capsids of serotype Asia I in silkworm larvae [12]. Because vaccination with the serotype Asia I vaccine does not protect cattle exposed to serotype A FMDV, it is necessary to develop an empty capsid subunit vaccine against this serotype. In the present study, we expressed the P1-2A and the 3C coding regions of a serotype A FMDV field isolate that caused outbreaks of FMD in China in 2009. These proteins were expressed in silkworm pupae (*Bombyx mori*) and assembled into empty capsids. Subsequent studies evaluated the ability of this serotype A FMDV empty capsid subunit vaccine to induce specific antibodies and protect cattle against homologous challenge after a single vaccination.

## Results

### Expression of Polyprotein in *Bm*-N Cells

**Figure 1:**
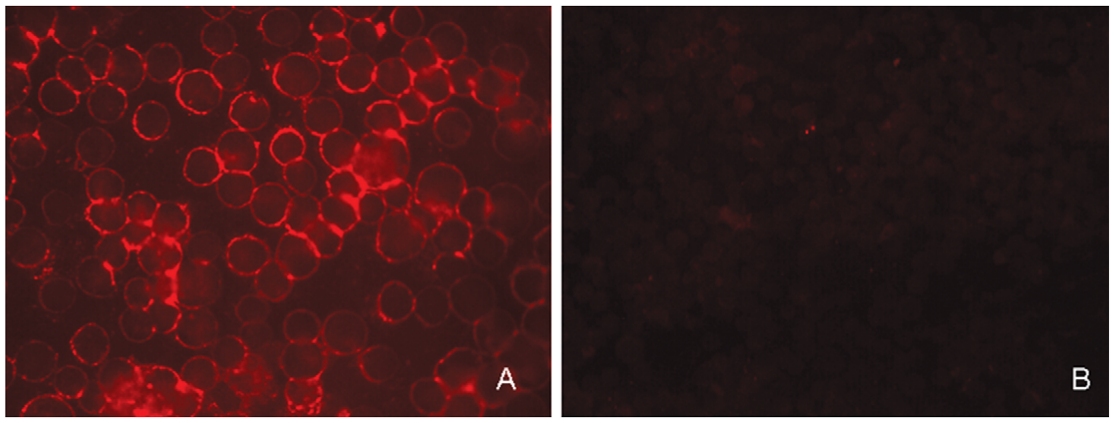
Expression of FMDV polypeptides in *Bm*-N cells analyzed by IFA. (A) *Bm*-N cells infected with rBmNPV (P1-2A3C). (B) *Bm*-N cells infected with BmBacPAK-6 (negative control).

Recombinant baculovirus rBmNPV (P1-2A3C) was generated by transfection of Bm-N cells with a recombinant bacmid that contained consecutive P1-2A and 3C coding regions of serotype A FMDV. The expression of the P1-2A3C polyprotein in *Bm*-N cells was analyzed by immunofluorescence assay (IFA). Fluorescence microscopy revealed that *Bm*-N cells infected with rBmNPV (P1-2A3C) produced specific fluorescence, while only very weak background fluorescence appeared in the uninfected control cells (Fig. 1). These data indicated that the P1-2A3C polyprotein was indeed expressed in *Bm*-N cell.

### Expression of Polyprotein in Silkworm Pupae

**Figure 2:**
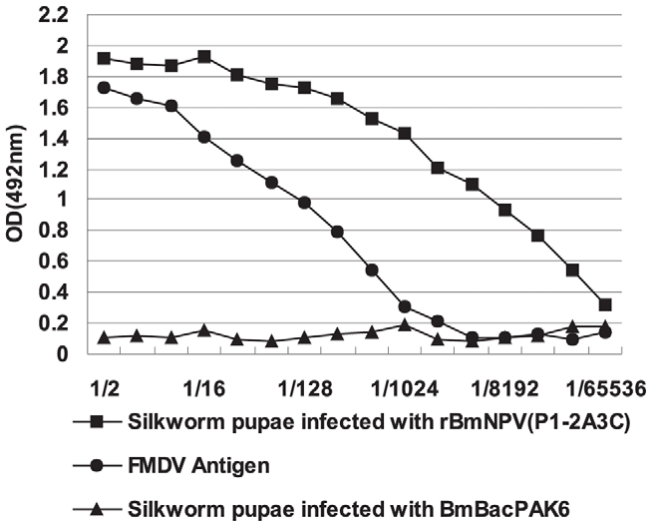
Expression of FMDV polypeptides in silkworm pupae estimated by sandwich-ELISA. Extracts from silkworm pupae were serially diluted two-fold.

**Figure 3:**
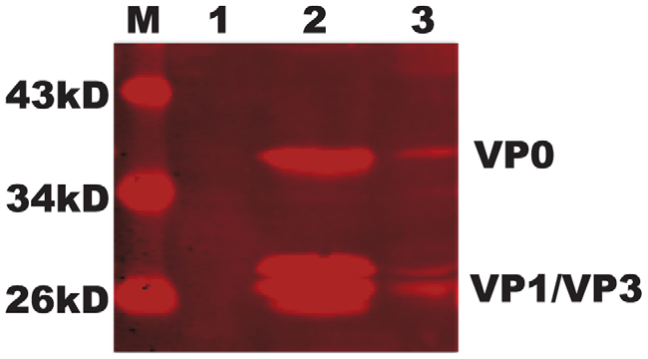
Western immunoblot analysis of purified empty capsid-like particles. Rabbit anti-FMDV polyclonal antibodies were used to detect empty capsid-like particles. Lane M, protein molecular weight marker; lane 1, purified products from silkworms infected with BmBacPAK-6 (negative control); lane 2, purified empty capsid-like particles from silkworms infected with recombinant baculovirus rBmNPV (P1-2A3C); lane 3, BHK-21 cell lysates infected with FMDV.

Similar to the FMDV antigen used as positive control, antigen-capture sandwich-ELISA indicated that the signal detected in the pupae infected with rBmNPV (P1-2A3C) decreased with dilution (Fig. 2). Furthermore, the expressed empty capsid-like particles in silkworm pupae were purified by gel filtration through a Sepharose 4 FF column. Western blot analysis of fractions was performed using polyclonal antibodies produced against FMDV. Processed capsid proteins, VP0 (36 kDa), VP1 (27 kDa), and VP3 (27 kDa), were identified (Fig. 3) and these proteins were not present in the negative control. These data demonstrated that recombinant P1-2A polyprotein was partially processed by the 3C protease.

### Observation of Immunogold-labeled FMDV Empty Capsid-like Particles

**Figure 4:**
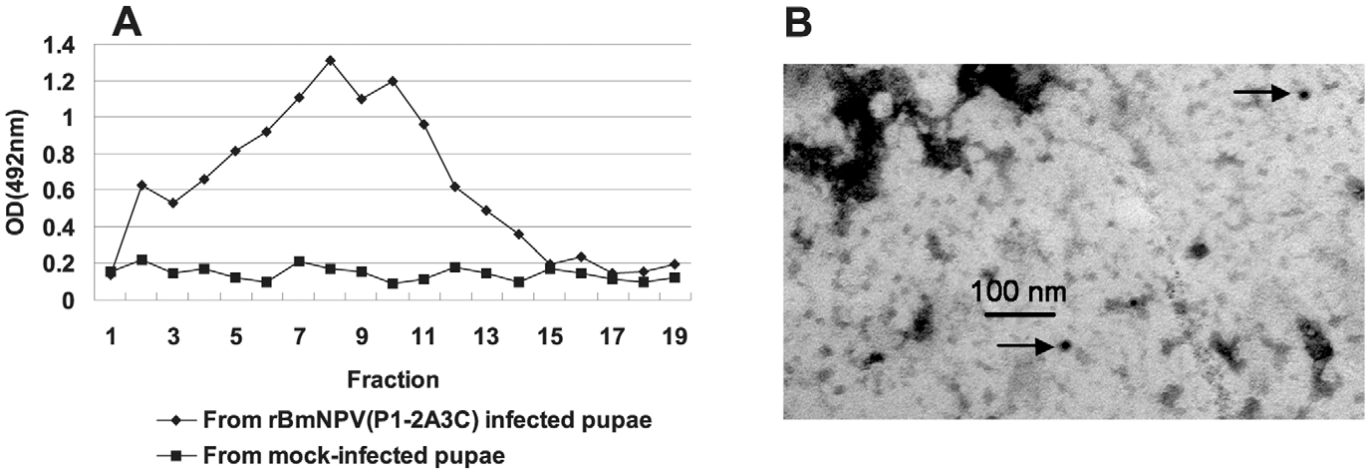
Electron microscopic analysis of immunogold labeled empty capsid-like particles. (A) Antigenic reactivity of individual fractions obtained after sucrose gradient centrifugation. The reactivity of each fraction was measured by sandwich-ELISA; (B) electron micrographs of FMDV empty capsid-like particles from rBmNPV(P1-2A3C)–infected pupae.

The expressed empty capsid-like particles were further purified by sucrose gradient centrifugation and collected fractions were analyzed by antigen-capture sandwich ELISA. The results revealed that the maximum amount of empty capsid- like particles was found in fractions 4–9 (Fig. 4A). From these fractions,fraction 8 was analyzed by staining with immunogold using FMDV-specific rabbit antiserum and 10 nm colloidal gold particle-conjugated secondary antibody. After negative staining, the empty capsid-like particles were viewed using an electron microscope (EM) and revealed spherical particles of approximately 30 nm diameter (Fig. 4B).

### Anti-FMDV Antibodies in Cattle

Cattle were vaccinated with extracts prepared from silkworm pupae infected with rBmNPV(P12A3C). As directed by the method recommended by the OIE/FAO World Reference Laboratory (WRL) for FMDV (Pirbright, UK), we evaluated the specific anti-FMDV antibody response by the liquid-phase blocking enzyme-linked immunosorbent assay (LPB-ELISA) [22], [23]. It was found that all five cattle vaccinated with rBmNPV (P1-2A3C) antigen developed a specific antibody response against FMDV at 14 days post-vaccination (dpv). By 21 and 28 dpv, the antibody titer maintained the same level or higher to a maximum titer of 180 in cattle No. 15 and No. 200. In contrast, the anti-FMDV antibody titer in the two control cattle (vaccinated with extracts from silkworm pupae infected with BmBacPAK-6) was not significant (Table 1).

**Table 1:** Analysis of cattle after challenge with FMDV.

Animal number	Vaccine^a^	LPB-ELISA antibody^b^ 0dpv	14dpv	21dpv	28dpv	Days of onset of pyrexia c	Duration of Pyrexia (days)	Lesion scores^d^	Protection^e^
7	rBm(P12A3C)	<8	90	128	128	−	−	−	+
15	rBm(P12A3C)	<8	128	256	180	−	−	−	+
25	rBm(P12A3C)	<8	45	90	90	−	−	−	+
200	rBm(P12A3C)	<8	128	128	180	−	−	−	+
195	rBm(P12A3C)	<8	16	32	22	Day2	3	4+mouth	−
5	BmBacPAK-6	<8	<8	<8	<8	Day2	3	4+mouth	−
32	BmBacPAK-6	<8	<8	<8	<8	Day2	3	4+mouth	−

a Cattle were vaccinated with extracts from silkworm pupae infected with rBmNPV(P1-2A3C) or the control (BmBacPAK-6) and challenged 28 days later.

b FMDV-specific antibody titer determined by LPB-ELISA.

c Pyrexia defined as body temperature ≥40°C.

d Lesion score is the number of feet on which the cattle exhibited.

e Protection was determined by no obvious FMD clinical signs during the observation period (10 days post-challenge).

### Challenge with FMDV A/WH/CHA/09

To test the effectiveness of the induced immune response to prevent FMDV infection, cattle were challenged at 28 dpv with 10,000 BID_50_ FMDV A/WH/CHA/09. Body temperature, mouth, and feet were observed consecutively for 10 days to evaluate the incidence of disease (Table 2). Only one vaccinated cow (No. 195) developed typical FMD lesions by 2 days post-challenge (dpc) and clinical signs were as severe as those observed in the control group. The remaining four vaccinated cattle that did not show clinical signs were considered completely protected. These data indicated that the P1-2A3C antigen produced in silkworm pupae elicited a protective immune response in cattle.

**Table 2:** FMDV-specific antibody response in the PD_50_ test.

Immunization dose of vaccine^a^	Animal number	LPB-ELISAantibody^b^ 14 dpv^c^	LPB-ELISA antibody^b^ 21 dpv^c^
1 dose of vaccine	1–1	180	180
	1–2	256	360
	1–3	90	90
	1–4	180	360
	1–5	180	180
1/3rd dose of vaccine	1–6	90	180
	1–7	45	90
	1–8	45	90
	1–9	90	180
	1–10	45	45
1/9th dose of vaccine	1–11	45	90
	1–12	32	32
	1–13	45	64
	1–14	32	32
	1–15	45	45
control group	1–16	<8	<8
	1–17	<8	<8

a Cattle were vaccinated with extracts from silkworm pupae infected with rBmNPV(P1-2A3C) or the control (BmBacPAK-6).

b FMDV-specific antibody titer reported as the serum dilution determined by LPB-ELISA.

c Days post-vaccination.

### PD_50_ Test

The LPB-ELISA antibody titer of vaccinated and control cattle was determined at 14 and 21 dpv using the LPB-ELISA. It was found that all of the 15 cattle vaccinated with extracts from silkworm pupae infected with rBmNPV(P1-2A3C) developed a detectable level anti-FMDV antibody response at 14 dpv, and some reached a titer of 256. By 21 dpv, the antibody level was maintained at the same level or higher. In contrast, the antibody level in control cattle was not significant (Table 2).

The potency of the vaccine was tested by challenging cattle at 21 dpv with 10,000 BID_50_ FMDV A/WH/CHA/09. Body temperature was recorded, and mouth and feet were examined for 10 days to determine the incidence of disease. Four of the five cattle were considered protected in the 1 dose immunized group, three of five cattle were protected in the 1/3rd dose group, and two of five cattle were protected in the 1/9th dose group. The vaccine potency of the batch immunized with the expressed P1-2A3C antigens reached 4.33 PD_50_ per dose (Table 3).

**Table 3:** PD_50_ test.

Immunization dose	Rate of protection (%)^a^	PD_50_ b
1	4/5	4.33
1/3	3/5	
1/9	2/5	

a Rate of protection (%) =  number of cattle with no lesions/total number of cattle.

b PD_50_ was calculated using the Karber method.

## Discussion

In March 2009, an FMDV serotype A outbreak in Wuhan, China resulted in the slaughter of approximately 9000 infected and susceptible animals. Prior to this outbreak there had been no previous outbreaks of serotype A FMDV in China and little research on a serotype A FMDV vaccine had been undertaken. Although vaccination with inactivated viruses has been shown to be efficient, it has been associated with several problems related to its safety and the discrimination between vaccinated and naturally infected animals [4]. Expression of empty capsids of the virus constitutes a feasible strategy to overcome both of these disadvantages [24]. In the present study, we expressed the FMDV capsid precursor protein and protease 3C in silkworm pupae (*Bombyx mori*). Production of FMDV empty capsid requires the proteolytic processing of P1 by protease 3C to generate the structural proteins VP0, VP1, and VP3, which self-assemble to form the viral capsid [25].

Veterinary products obtained using gene expression techniques are typically constrained by the final price of the antigen due to production costs. Thus, the production of a safe, low-cost source of antigens is required for feasible vaccine design. The silkworm expression system is an innovative and simple approach to achieve high protein expression levels [19]. In a previous report, we assembled empty capsids of FMDV serotype Asia I using silkworm larvae as a bioreactor [12]. However, the baculovirus expression system using silkworm larvae as a bioreactor is not always suitable for year-round production. The rearing of silkworm larvae is limited by the season and the conditions, such as the supply of mulberry leaves. Silkworm pupae have also been used as a bioreactor for recombinant protein production [26], [27] and since they have prolonged survival at 2–8°C and do not require mulberry leaves or an artificial diet during their growth, silkworm pupae can be used to express proteins of interest at any time throughout the year. Furthermore, the expressed product can be directly purified from the infected pupae without any of the drawbacks associated with larvae. Thus, silkworm pupae are more convenient and economical than silkworm larvae for the expression of foreign proteins. In the present study, we used silkworm pupae as a bioreactor to express the capsid precursor protein and protease 3C of FMDV A/WH/CHA/09. Sandwich-ELISA showed that the quantity of FMDV protein expressed in silkworm pupae was much higher than that obtained from FMDV infected cells *in vitro*.

Extracts of silkworm pupae infected with rBmNPV(P1-2A3C) were used to vaccinate five cattle and two controls. The anti-FMDV antibody response developed in cattle was evaluated using the LPB-ELISA at 14dpv, 21dpv and 28 dpv. The LPB-ELISA has previously been proved to correlate well with the standard virus neutralization test (VNT) for measuring the serum antibody titers of FMD vaccinated animals [22], [23]. According to unpublished data obtained in our laboratory, approximately 50% of vaccinated cattle are protected from virus challenge when the LPB-ELISA antibody titers are 45–90. The current study revealed that the antibody titers of four vaccinated cattle reached at least 90 at 28dpv (Table 1). Only one vaccinated animal (No. 195) developed a low-level antibody titer that proved to not be protective. These data indicate that the expressed products from silkworm pupae were immunogenic and the immune response elicited was protective. After virulent homologous virus challenge, four of the five vaccinated cattle were protected and the remaining animal developed lesions on all the feet and in the inside of the mouth on the second day, similar to unvaccinated control cattle. Thus, protection from challenge was consistent with LPB-ELISA antibody titers.

Using the data obtained from challenge experiments, the bovine potency test protocol described by the OIE was used to evaluate the efficacy of the empty capsid subunit vaccine. The empty capsid subunit vaccine potency was determined to be 4.33 PD_50_ per dose for cattle. Thus, when employed for routine prophylactic use, the vaccine should contain at least 3 PD_50_ per dose for cattle as recommended by the OIE. In conclusion, the use of silkworm pupae (*Bombyx mori*) as bioreactors is feasible for the expression of immunogenic proteins to develop a next generation FMDV empty capsid subunit vaccine.

## Materials and Methods

### Ethics Statement

All the experiments involving animals were approved by the animal ethics committee of Lanzhou Veterinary Research Institute (Permit Number: 09–26) and followed the national guidelines for the use of animals in scientific research.

### Viruses and Cell Lines

FMDV A/WH/CHA/09 strain was propagated in the BHK-21 cell line which is extensively used for the diagnosis and identification of FMDV [28], isolated, and preserved at Lanzhou Veterinary Research Institute of the Chinese Academy of Agriculture Sciences. The parental virus BmBacPAK-6 (Chinese patent: 1242428), *Bm*-N cell line, and silkworm variety JY1 were maintained at the Biotechnology Research Institute of the Chinese Academy of Agriculture Sciences. The BmBacPAK-6 and recombinant viruses were maintained in *Bm*-N cells at 27°C in TC-100 insect medium (Invitrogen, USA) supplemented with 10% heat-inactivated fetal bovine serum (Invitrogen, USA).

### Construction and Screening of Recombinant Baculovirus

Viral RNA was extracted from cell culture supernatants using RNeasy (Qiagen, Germany) and used immediately for cDNA synthesis. The cDNA was synthesized using AMV reverse transcriptase and Oligo(dT)_18_ primer (Takara, China) at 42°C for 1 h following the recommended protocol. PCR was used to amplify the intact P1-2A and 3C protease coding regions from the cDNA using two pairs of specific primers: forward primer for P1-2A, 5′-ATAAGATCTACCATGGGGGCAGGGCAATCTAGCC-3′ (*Bgl*II restriction site underlined); reverse primer for P1-2A, 5′-GCGTCTAGATGACATGTCCTCTTGCATCTGGTTA-3′ (*Xba*I restriction site underlined); forward primer for 3C, 5′-GCGTCTAGAAAGAAACCTGTCGCTTTGAAAGT-3′ (*Xba*I restriction site underlined); reverse primer for 3C, 5′-ATAAGATCTCTACTCGTGGTGTGGTTCGGGAT-3′ (*Bgl*II restriction site underlined). The target fragments of P1-2A and 3C were sequentially cloned into *Bam*HI/*Xba*I and *Xba*I/*Bgl*II sites, respectively, of the baculovirus transfer vector, pVL1393, and named pVL-P12A3C. The pVL-P12A3C baculovirus transfer plasmid was co-transfected with linearized Bm-BacPAK6 DNA into *Bm*-N cells via a liposome-mediated method [29]. The co-transfection supernatant was subjected to three rounds of plaque assays to screen individual viral plaques.

### Expression of FMDV Polyprotein in *Bm*-N Cells

The expression of FMDV P1-2A3C polyprotein in *Bm*-N cells infected with rBmNPV(P1-2A3C) was analyzed by IFA. The rBmNPV (P1-2A3C) was multiplied in *Bm*-N cells. *Bm*-N cells (2.0×10^5^) were cultured on cover slips and inoculated with Bm-P12A3C at a multiplicity of infection (MOI) of 10. At 48 h post-infection (hpi), IFA was conducted to analyze the expression of FMDV proteins. Cells were then rinsed twice phosphate-buffered saline (PBS) and fixed in 100% cold acetone (−20°C for 30 min). Samples were incubated with rabbit anti-FMDV serum (37°C for 30 min) in a humid box, washed five times with PBS, and then stained with fluorescein-conjugated goat anti-rabbit serum at 37°C for 30 min. Cover slips were coated with glycerin and observed on a fluorescence microscope. *Bm*-N cells infected with BmBacPAK-6 were used as negative controls for these analyses.

### Expression of P1-2A3C Polyprotein in Silkworm Pupae

The silkworm pupae were infected with the recombinant virus at approximately 10^5^ plaque-forming units (pfu) per pupae. Approximately 50 g of silkworm pupae infected with rBmNPV(P1-2A3C) were homogenized in 50 mL of homogenization buffer (100 mM PBS, pH 7.6) using a polytron PT2100 homogenizer (Kinematica, Luzern, Switzerland) at 20,000 rpm. The homogenate was clarified by centrifugation at 10,000×*g* for 15 min at 4°C. After removal of the lipid layer with a spatula, the supernatant was collected on ice and stored at −20°C. An antigen-capture sandwich enzyme-linked immunosorbent assay (ELISA) and western immunoblot were used for detection of P1-2A3C polyprotein.

The antigen-capture sandwich ELISA was carried out as described by the World Organization for Animal Health, Office International desEpizooties (OIE) standard for FMDV serotyping. In brief, microtiter plates were coated with anti-FMDV type A rabbit sera at a dilution of 1∶1000 in carbonate coating buffer overnight at 4°C. All subsequent incubations were performed at 37°C for 1 h, using 50 µL of reagent added to each well. The extracts of silkworm pupae infected with rBmNPV(P1-2A3C) were serially diluted two-fold and added to the wells, and inactivated FMDV A/WH/CHA/09 virions and mock-infected antigen were added as positive and negative controls, respectively. Plates were incubated with anti-FMDV serotype A guinea pig sera, followed by horseradish peroxidase-conjugate rabbit anti-guinea pig IgG (Sigma, USA) at 1∶10,000 dilution. Substrate (0.05% H_2_O_2_ plus orthophenylene diamine) was added, allowed to react for 15 min and stopped by the addition of 1M H_2_SO_4_. The absorbance at 492 nm was determined for each well.

For western immunoblot analyses, the harvested supernatant was diluted with five volumes of PBS (100 mM, pH 7.6) and passed through a 0.45 µm filter (Pall, USA). The filtrate was then purified by gel filtration using a Sepharose 4 Fast Flow (FF) column (GE Healthcare, USA) with elution buffer (100 mM Tris-HCl, pH 8.0). The first elution peak containing the empty capsid-like particles was collected and separated by 10% sodium dodecyl sulfate-polyacrylamide gel electrophoresis (SDS-PAGE). The separated proteins were electro-transferred onto a nitrocellulose membrane, blocked, and then incubated with rabbit FMDV antiserum. Rabbit primary antibodies were detected using IRDye 700DX-conjugated anti-rabbit antibody (Rockland Immunochemicals, USA), and the membrane was scanned using the LiCor Odyssey fluorescent scanning system (LiCor Biosciences, USA).

### Observation of Immunogold Labeled FMDV Empty Capsid-like Particles

The elution peak containing empty capsid-like particles were layered onto a 15–45% (w/v) sucrose gradient in NET buffer (100 mM NaCl, 1 mM EDTA, 50 mM Tris-HCl; pH 7.5) and centrifuged at 35,000×*g* for 2.5 h at 4°C. The resulting gradients were collected in 20 500 µL-fractions and analyzed by antigen-capture sandwich ELISA. Fractions containing empty capsid-like particles were collected and stored at 4°C for subsequent analysis. For immunogold labeling of the empty capsid-like particles, 20 µL of each fraction was placed onto grids and incubated for 5 min. Grids were blocked with 3% bovine serum albumin in PBS for 30 min and incubated with anti-FMDV type A rabbit sera for 30 min at room temperature. Grids were then washed three times with PBS and incubated with gold-conjugated secondary antibodies (10-nm gold-conjugated anti-rabbit antibody) for 30 min at room temperature. After secondary-antibody incubation, grids were washed with PBS three times, followed by negative staining with 1% phosphotungstic acid. Transmission electron micrographs were captured.

### Vaccination of Cattle

Silkworm pupae extracts were prepared by homogenizing 50 g of silkworm pupae infected with rBmNPV(P1-2A3C) in 250 mL PBS mixed with Montanide ISA 206 oil adjuvant. In order to determine the antibody titer for screening of candidate cattle for vaccination, liquid-phase blocking (LPB)-ELISA was performed according to the standard OIE method (http://www.oie.int/eng/norms/MMANUAL/A_00024.htm). A detection kit was prepared by the National Foot and Mouth Disease Reference Laboratory (OIE Reference laboratory for Foot and Mouth Disease), Lanzhou Veterinary Research Institute. Cattle with an antibody titer <8 were housed in the animal biosafety level (ABSL)-3 laboratory at Lanzhou Veterinary Research Institute. Five cattle were immunized (2 mL per cattle) by intramuscular inoculation in the neck with extracts of silkworm pupae infected with rBmNPV(P1-2A3C). Two control cattle were vaccinated with the same dose of extracts from silkworm pupae infected with Bm-BacPAK6. Cattle sera were collected at 14, 21, and 28 dpv. Anti-FMDV antibody was detected using the LPB-ELISA, as previously described.

### Challenge with Virulent Homologous FMDV

According to the standard protocol described by the OIE (http://www.oie.int/eng/norms/MMANUAL/A_00024.htm), all animals were challenged at 28 dpv by intradermal inoculation at two sites in the tongue with 10,000 bovine infectious doses (BID_50_) of A/WH/CHA/09. The body temperature of the animals was monitored daily. Restrained animals were carefully examined in the mouth and feet every day for the first 10 dpc.

### PD_50_ Test

The OIE PD_50_ test was performed to test the potency of the empty capsid subunit vaccine. The study consisted of three groups of five cattle were vaccinated and a control group of two non-vaccinated animals. The vaccinated groups were immunized with a full dose (2 mL), 1/3th dose (0.66 mL) or 1/9th dose (0.22 mL) of the empty capsid subunit vaccine. Cattle sera were collected at 14 and 21 dpv, and determined by LPB-ELISA. All animals were challenged 21 dpv with 10,000 BID_50_ of A/WH/CHA/09 administered by intradermal inoculation into two sites on the upper surface of the tongue. The cattle were observed daily for clinical signs of FMD for 10 dpc. Control cattle developed lesions on at least three feet. Unprotected animals showed lesions at sites other than the tongue. The PD_50_ of the vaccine was calculated based on the Karber method.
